# Phenotypic analysis of images of zebrafish treated with Alzheimer's γ-secretase inhibitors

**DOI:** 10.1186/1472-6750-10-24

**Published:** 2010-03-22

**Authors:** Dilyara Arslanova, Ting Yang, Xiaoyin Xu, Stephen T Wong, Corinne E Augelli-Szafran, Weiming Xia

**Affiliations:** 1 Center for Neurologic Diseases, Department of Neurology, Brigham and Women's Hospital, Harvard Medical School, Harvard University, Boston, MA, USA; 2 Department of Radiology, Brigham and Women's Hospital, Harvard Medical School, Harvard University, Boston, MA, USA; 3 Department of Radiology, The Methodist Hospital Research Institute, Weill Medical College of Cornell University, Houston, TX, USA

## Abstract

**Background:**

Several γ-secretase inhibitors (GSI) are in clinical trials for the treatment of Alzheimer's disease (AD). This enzyme mediates the proteolytic cleavage of amyloid precursor protein (APP) to generate amyloid β protein, Aβ, the pathogenic protein in AD. The γ-secretase also cleaves Notch to generate Notch Intracellular domain (NICD), the signaling molecule that is implicated in tumorigenesis.

**Results:**

We have developed a method to examine live zebrafish that were each treated with γ-secretase inhibitors (GSI), DAPT {N- [N-(3,5-Difluorophenacetyl-L-alanyl)]-S-phenylglycine *t*-Butyl Ester}, Gleevec, or fragments of Gleevec. These compounds were first tested in a cell-based assay and the effective concentrations of these compounds that blocked Aβ generation were quantitated. The mortality of zebrafish, as a result of exposure to different doses of compound, was assessed, and any apoptotic processes were examined by TUNEL staining. We then used conventional and automatic microscopes to acquire images of zebrafish and applied algorithms to automate image composition and processing. Zebrafish were treated in 96- or 384-well plates, and the phenotypes were analyzed at 2, 3 and 5 days post fertilization (dpf). We identified that AD95, a fragment of Gleevec, effectively blocks Aβ production and causes specific phenotypes that were different from those treated with DAPT. Finally, we validated the specificity of two Notch phenotypes (pigmentation and the curvature of tail/trunk) induced by DAPT in a dose-dependent manner. These phenotypes were examined in embryos treated with GSIs or AD95 at increasing concentrations. The expression levels of Notch target gene *her6 *were also measured by *in situ *hybridization and the co-relationship between the levels of Notch inhibition by DAPT and AD95 and the severity of phenotypes were determined.

**Conclusion:**

The results reported here of the effects on zebrafish suggest that this newly developed method may be used to screen novel GSIs and other leads for a variety of therapeutic indications.

## Background

High throughput screening in invertebrate animals has emerged as a powerful tool for drug discovery, but whole vertebrate animal-based high throughput screening has yet to be developed and refined. The zebrafish is one of the most cost-effective vertebrates that can be used for high throughput and high content screens. Phenotype-based small molecule screening in zebrafish has been described in a number of studies [1]. One successful screen used a previously characterized mutant zebrafish, *Gridlock*, that is defective in aortic blood flow reminiscent of aortic coarctation in humans [2]. A library of 5,000 small molecules was applied to *Gridlock *embryos, followed by manual examination using fluorescence microangiography. Two small molecules were identified to suppress the *Gridlock *phenotype in a dose-dependent manner [3]. For non-fluorescent zebrafish, we have developed algorithms to analyze certain morphological changes in the development of zebrafish somites [4]. These changes in morphology were linked to the lack of a component of the γ-secretase [5], the key protease involved in the pathogenesis of Alzheimer's disease (AD) [6].

AD is a progressive neurodegenerative disorder that is pathologically characterized by the presence of extracellular and intracellular lesions known as amyloid plaques (extracellular) and neurofibrillary tangles (intracellular) [6]. Amyloid plaques are formed by the accumulation of amyloid β (Aβ), a 4 kDa peptide that is generated by sequential cleavage of amyloid precursor protein (APP) by β-secretase and γ-secretase [6,7]. γ-Secretase is an aspartyl protease that mediates the final cleavage to generate Aβ at residue 40 (Aβ40) or 42 (Aβ42). It is composed of presenilins (PS1 or PS2), presenilin enhancer (Pen-2), nicastrin, and Aph-1 [8-10]. PS carry the active site of the γ-secretase [11], and missense mutations in PS genes account for majority of early onset familial AD cases. γ-Secretase has also been identified as the key protease involved in the pathogenesis of certain types of cancers, such as leukemia [12].

Inhibiting the production of Aβ by targeting γ-secretase constituents is an attractive approach for developing new treatments of AD, but has potential toxic side effects. Finding inhibitors of γ-secretase complex to simply block Aβ production is no longer a challenge, and a number of potent γ-secretase inhibitors (GSIs) have been published [13]. However, inhibiting γ-secretase not only prevents APP cleavage and Aβ production, but also blocks the cleavage of other important proteins. γ-Secretase cleaves dozens of other type I transmembrane proteins that are critically involved in many metabolic pathways, including Notch [14]. When both PS1 and its homolog PS2 are knocked out, the resulting phenotype is indistinguishable from the Notch knockout, and no Aβ is produced [15]. In mice, Notch deficiency produces embryonic-lethal phenotypes, demonstrating the essential nature of Notch signaling in embryonic development.

Notch signaling plays an important role in health and disease states during embryonic development throughout adulthoods. γ-Secretase cleaves Notch encoded transmembrane receptor and activates signal transduction by releasing the Notch intracellular domain (NICD) [16]. NICD controls transcription of *hairy *and *enhancer of split *genes, such as *hes1 *in mammals [17] and *her6 *in zebrafish [18,19]. *Hes *genes are essential effectors of Notch signaling that regulate the maintenance of undifferentiated cells [20]. In rats lacking *Hes1, Hes3*, or *Hes5*, all neural stem cells prematurely differentiate into neurons without generating glial cells. This causes a wide range of brain defects in size, shape and cell organization [21]. In zebrafish, Notch is extremely important in embryonic development and is one of the driving forces behind neural crest cell differentiation. Inhibition of Notch signaling leads to extensive defects in organs derived from neural crest cells. The two most prominent phenotypes in zebrafish are somitogenesis and pigmentation. These phenotypes are illustrated in many zebrafish Notch pathway mutants, such as *bea, des, aei*, and *wit *[22,23]. Treating zebrafish with a potent γ-secretase inhibitor (GSI), DAPT, at the late blastula stage causes defects in somitogenesis and neurogenesis [24]. Similarities have been observed between DAPT-treated embryos and Notch pathway mutants, and certain phenotypes in DAPT treated embryos can be partially rescued by microinjection of NICD mRNA [24].

The implication of Notch signaling in cancers arose from the observation that more than 50% of human T-cell acute lymphocytic leukemia (T-ALL) has activating mutations in Notch1 gene. This results in constitutively active Notch signaling [25]. Perturbed Notch signaling has been associated with different types of tumor cell lines. It has been found that γ-secretase inhibitors suppress these tumorigenesis pathways by reducing Notch signaling [26-28]. Interestingly, a recent study found that a combination of enhanced Notch signaling and Abelson leukemia (Abl) tyrosine kinase activity promotes acute lymphocytic leukemia [29]. Gleevec has been approved for the treatment of chronic myeloid leukemia and gastrointestinal stromal tumors, and mechanistically, Gleevec binds to Abl tyrosine kinase and locks the kinase in an inactive conformation by interacting with aspartate and phenylalanine [30-32]. In addition, Gleevec was shown to selectively inhibit APP cleavage and Aβ production at high concentrations [33], in contrast to DAPT that blocks both APP and Notch cleavages. Therefore, the Gleevec molecule seems to contain functionalities that cause inhibition of Abl tyrosine kinase and γ-secretase independently. The co-crystal structure of the Abl tyrosine kinase domain and Gleevec shows that the pyrimidine and pyridine rings of the Gleevec overlap with the ATP-binding site via Thr315 [30-32]. The goal of this study is to dissect the structure of Gleevec and examine the effects of each fragment both *in vitro *and *in vivo *on γ-secretase cleavage of APP and Notch in cultured cell- and whole animal-based assays. To facilitate data collection, we developed a semi-automated method for embryo imaging and phenotype recording. This approach can be used for future development of high throughput screening of novel GSIs and other therapeutic leads for the treatment of AD, leukemia, and other diseases.

## Methods

### A. Embryo Treatment

Embryos were placed in a 24-well plate (5-6 embryos/well). Compounds were dissolved in 1 mL of egg water (final concentration at 50 μM for DAPT {N- [N-(3,5-Difluorophenacetyl-L-alanyl)]-S-phenylglycine *t*-Butyl Ester}, Gleevec, AD28, AD94, AD95, and 10 μM for AD115; 0.1% DMSO was used as a negative control). Embryo medium was replaced with the compound containing egg water, and the embryos were incubated at 28°C overnight before survival rate was recorded and photographic images were taken. Compounds were applied at two time points, 6 hours and 24 hours post-fertilization (hpf). Prior to the treatment at 24 hpf, embryos were de-chorionated in pronase [34]. Embryos treated at 6 hpf were de-chorionated the following day after the compound treatment.

### B. Blocking Aβ production by γ-secretase inhibitors

Compounds were dissolved in the DMEM growth media and applied to ~90% confluent APP-expressing CHO cells in 96-well plates. After incubation for 4-5 hours at 37°C, cells were centrifuged for 5 minutes at 6000 g, and supernatant media were collected for Aβ measurement by ELISA. Sandwich ELISAs for monomeric Aβ were performed as described [35,36]. The capture antibodies 2G3 (to Aβ residues 33-40) and 21F12 (to Aβ residues 33-42) were used for Aβ40 and Aβ42 species. The detecting antibody was biotinylated 266 (to Aβ residues 13-28). These antibodies were kindly provided by Dr. P. Seubert and Dr. D. Schenk (Elan, plc).

### C. TUNEL Staining

To examine the apoptotic effect of the compounds on embryos, TUNEL staining was performed (*In Situ *Cell Death Detection Kit, TMR red, Roche). Embryos fixed in 4% paraformaldehyde overnight at 4°C, de-chorionated, and treated with 1% Triton-X100 in PBS. The reaction was carried out at 37°C for 1 hour, and all staining procedures were performed per the instruction by the manufacturer [5]. The embryos were mounted in 3% methylcellulose and images were acquired under 51× magnification.

### D. In situ Hybridization

*In situ *hybridization was carried out according to standard protocols [37] using the *her6 *probe. Single-stranded RNA probes against *her6 *were synthesized from a cDNA clone (kindly provided by Dr. P Raymond, University of Michigan, Ann Arbor, MI) using T7 RNA polymerase after linearization by restriction digest, then labeled with digoxigenin-UTP (Roche, Basel, Switzerland). At least 10 to 20 embryos were examined in each experiment.

To quantify the expression levels of Notch downstream gene her6, we used manual approach to segment the her6-positive brain regions from the dorsal view images. The region of interest was manually segmented, and the intensity of each ROI was quantified. The levels of *her6 *expression in DMSO treated embryos were normalized to 1, and relative levels of other embryos were calculated.

### E. Conventional Microscope Imaging

Compound-treated embryos were observed under an OLYMPUS SZX12 microscope. For examination, embryos were removed from the compound-containing medium and placed in 0.4% tricane (3-amino benzoic acid ethyl ester, Sigma, St. Louis, MO) solution. Upon anesthetizing, embryos were placed in 3% methylcellulose for positioning and images were recorded with an OLYMPUS Q-COLOR3 camera. Images were taken at 51× magnification for embryos at 2 dpf, and 40× magnification for embryos at 4 and 6 dpf.

### F. Automatic Imaging by In Cell Analyzer 1000

Embryos were treated with compounds at two time points (6 hpf and 24 hpf) in the 96- or 384-well plates (1 embryo/well) with compound-containing media (final concentration at 50 μM for all compounds except for 10 μM of AD115). For image acquisition, tricane was added to the final concentration of 0.4% in the same plate, and the plate was loaded into In Cell Analyzer 1000 (General Electric Co., Fairfield, CT). Images of individual wells were automatically acquired by In Cell Analyzer 1000. From each well of the plate, 4 z-series were scanned from top to bottom, and 4-6 fields of each well were taken. Complete coverage of individual wells of the microtiter plate usually resulted in poor resolution and prevented further image processing. On average, ~10 minutes were needed for the acquisition of images from a 384-well plate, and there was no effect on the development of zebrafish during this process. Images acquired by In Cell Analyzer 1000 were processed using MATLAB (Mathworks, Natick, MA), and fields from each well were automatically merged using "Merge" function from Adobe Photoshop Elements (Adobe Systems Inc., San Jose, CA). Using these approaches, the average time used for automatic image acquisition and processing was significantly reduced compared to conventional microscope-based image acquisition, which would require approximately 10-20 fold more time to analyze the same number of animals. Pigmentation was quantified in a semi-automatic approach. First, manual drawing of region of interest (ROI) on an image was carried out, followed by calculation of the optimum threshold separating the ROI into two classes, pigmentation and background [38]. The advantage of this method is that it minimizes the combined intra-class variances with a moderate computational complexity. However, the method may generate false-positive and false-negative due to uneven illumination and noise in the measurement. To circumvent this problem, a size-filter was used to discard small segmented areas when the areas are less than a small positive threshold α based on the prior knowledge that true pigmentation is usually larger than α pixels. In this work we set α to 10. After segmenting the pigmentation, the total area and average pixel values of pigmentation were computed. The values of the normal control group were used as the benchmark for comparison. If the average pixel density of an image was less than the control group, it was marked as 'lighter'.

### G. In Vitro γ-Secretase Cleavage of Notch Substrate

The *E. coli *generated Notch-based, 100-residue γ-secretase substrates N100-Flag were purified as previously described [39]. N100-Flag substrate contains an initiating methionine, 99 amino acids that start at the TACE cleavage site, and a Flag tag. Compounds were mixed with solubilized γ-secretase complex and the in vitro γ-secretase cleavage of Notch substrate was carried out as previously described [40].

## Results

### A. Reduction of Aβ generation by fragments of Gleevec

**Figure 1:**
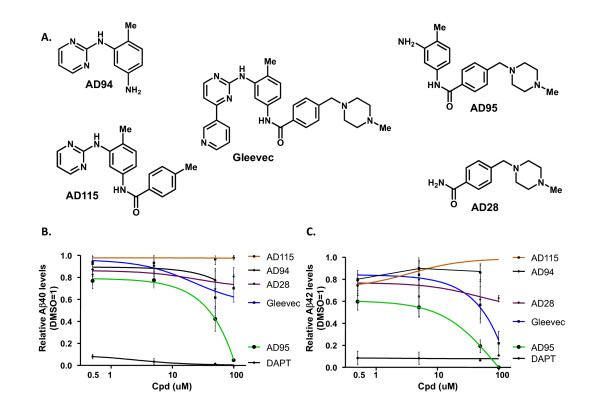
**Inhibition of Aβ production in cultured cells by fragments of Gleevec**. A. Structures of Gleevec and its fragments used for the treatment of cultured cells and zebrafish embryos. Four fragments of Gleevec are named as AD28, AD94, AD95 and AD115. B and C. Inhibition of γ-secretase cleavage of APP was determined by measuring the levels of Aβ40 (B) and Aβ42 (C) in the conditioned-media by ELISA. Levels of Aβ from cells treated with 0.1% of DMSO were normalized to 1 and used to calculate the relative levels of Aβ from cells treated with DAPT, Gleevec, and its fragments. Standard errors of means are illustrated by bars.

Three types of compounds were used to treat zebrafish embryos: DAPT (a well-established, potent GSI), Gleevec (a reported APP-selective GSI), and the fragments of Gleevec. Four individual Gleevec fragments were synthesized and designated as AD28, AD94, AD95, and AD115 (Fig. 1A). An APP overexpressing CHO cell line 7W was treated with individual compounds and the levels of secreted Aβ in the media were measured by ELISA. Levels of Aβ in the media of 7W cells that were treated with DMSO were normalized to 1, and relative levels of Aβ40 (Fig. 1B) and Aβ42 (Fig. 1C) were calculated. As expected, cells treated with the potent GSI, DAPT, showed a strong inhibition of the generation of Aβ, even at the concentration of 0.5 μM. At high concentration of 50 μM, both Gleevec and AD95 showed inhibition of Aβ production, while AD28 and AD94 showed less inhibition. AD115 failed to inhibit Aβ production even at 100 μM (Fig. 1B, C).

### B. Toxicity of GSI in zebrafish embryos

We routinely generated large clusters of zebrafish embryos and treated several dozen embryos with each compound. To assess the effects of these compounds on the zebrafish survival rate, we used a conventional microscope and quantified embryo survival rate at 1 and 2 dpf (Table 1). When the concentrations of these compounds were at 50 μM, the survival rate of embryos treated with each of the compounds was close to 100% except for AD115. None of the embryos treated with 50 μM AD115 survived. When the concentration of AD115 was reduced to 10 μM, all embryos survived at 1 dpf, and about 90% survived at 2 dpf.

**Table 1:** Survival rate of the compound treated embryos.

Cpd	Conc.	Alive/Total Embryos	Alive/Total Embryos
		**Day 1**	**Day 2**

DMSO	0.10%	35/37	34/35

DAPT	50 μM	32/33	30/32

Gleevec	50 μM	48/50	47/48

AD28	50 μM	28/28	27/28

AD94	50 μM	15/18	15/15

AD95	50 μM	17/18	17/17

AD115	50 μM	0/10	0/0

AD115	10 μ;M	33/34	30/33

**Figure 2:**
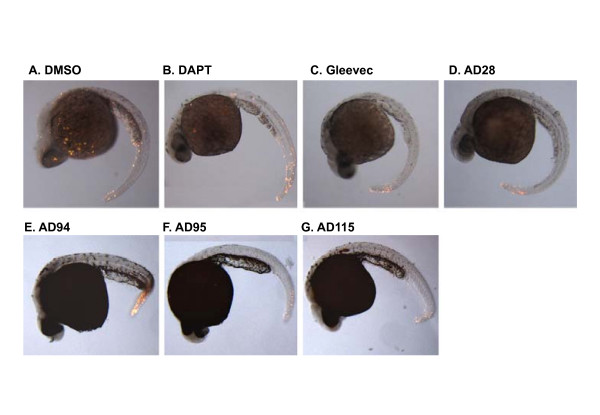
**Apoptosis in zebrafish embryos treated with compounds**. Zebrafish embryos were treated with GSI or fragments of Gleevec at 6 hpf. Embryos were then fixed and subjected to TUNEL staining at 24 hpf. Control embryos were treated with 0.1% DMSO and the remaining embryos were treated with different GSIs or Gleevec fragments at 50 μM, except for AD115 whose concentration was 10 μM. The majority of apoptotic cells were observed in the fish tail, but no difference among individual compound-treated embryos was observed. The representative images from 2 different experiments are presented.

To determine whether these compounds induced regional apoptosis in zebrafish embryos, apoptotic cell death was assessed via TUNEL staining (Fig. 2). Embryos were treated with 50 μM of compounds (except AD115 was at 10 μM), and TUNEL staining of these embryos were compared to that of 0.1% DMSO-treated embryos. While DMSO treated embryos showed a few apoptotic cells throughout their bodies (Fig. 2A), compound-treated embryos showed some apoptotic cells mostly in the tail region. AD28 (Fig. 2D), AD95 (Fig. 2F), and AD115 (Fig. 2G) showed a comparable number of apoptotic cells to Gleevec-treated embryos (Fig. 2C). AD94 showed a slightly higher number of apoptotic cells in the tail compared to the other embryos (Fig. 2E), and DAPT did not induce extensive apoptosis in embryos compared to DMSO-treated embryos (Fig. 2B).

### C. Compound Induced Phenotypes in Zebrafish Embryos

**Figure 3:**
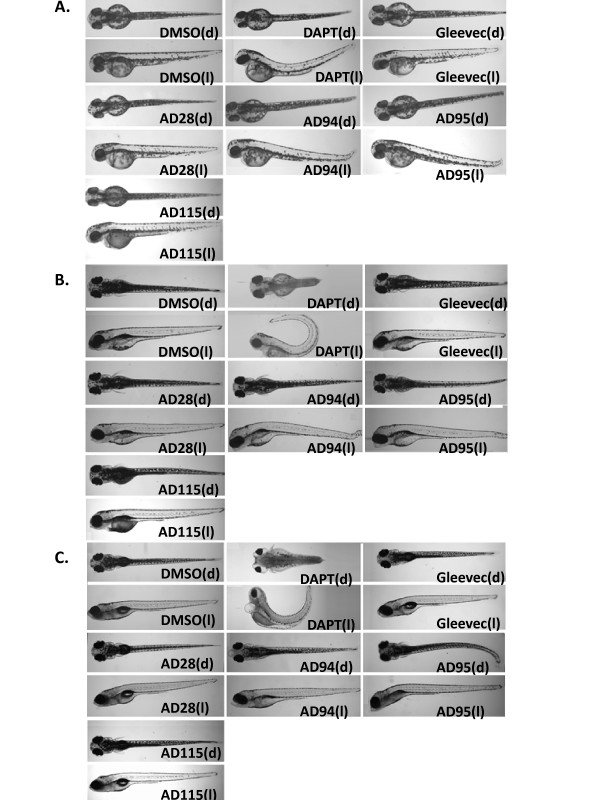
**Phenotypes of the embryos treated with GSI, Gleevec, and fragments at 24 hpf**. Dorsal view (indicated as "d", top panel) and lateral view (indicated as "l", bottom panel) of embryos are shown. Control embryos treated with 0.1% DMSO show a wild-type phenotype. Embryos treated with GSI, DAPT, resulted in defects in somitogenesis, eye development, pigmentation, and trunk formation as observed at 2 dpf (A), 4 dpf (B), and 6 dpf (C). Embryos treated with DAPT, AD94 or AD95 started to show defects at 2 dpf, and phenotypes of remaining embryos were most obvious at 4 or 6 dpf. The following concentrations were used: 50 μM DAPT, 50 μM Gleevec, 50 μM AD28, 50 μM AD94, 50 μM AD95, and 10 μM AD115.

The zebrafish embryos treated with different compounds were examined every 1-2 days up to 6 dpf. Images were acquired by two methods using conventional microscope and microscopic instrument In Cell Analyzer. Morphological phenotypes of zebrafish embryos treated with DAPT, Gleevec or fragments of Gleevec by conventional microscope were compared first to document these phenotypes (Fig. 3). Images of compound-treated embryos were acquired at 2 dpf (Fig. 3A), 4 dpf (Fig. 3B) and 6 dpf (Fig. 3C). The DMSO-treated embryos showed a wild-type phenotype and developed normally. Embryos developed normal eyes, straight trunk and tail (Fig. 3A), V-shaped somites (Fig. 3B and 3C), and normal pigmentation (6 dpf) (Fig. 3C). Embryos treated with DAPT, the potent GSI known to inhibit both APP and Notch processing, showed curved tail, a bent trunk (Fig. 3A, B), smaller eyes, disturbed somites, and a decrease in pigmentation (Fig. 3C). Almost all Gleevec-treated embryos developed normally with a phenotype similar to the wild-type embryos. Only 1 out of 40 Gleevec-treated fish showed smaller size, and 4 out of 40 showed a shorter tail. AD28-treated embryos showed normal pigmentation and V-shaped somites with the phenotypes similar to wild-type embryos. Less than 20% (5 out of 28) embryos showed short, slightly curved tails (Table 2).

**Table 2:** The phenotypes of compound-treated zebrafish imaged by conventional microscope.

Cpd	Conc.	Size	Eye	Pigment	Tail	Trunk
DMSO	0.10%	normal	normal	normal	all straight	straight

DAPT	50 μM	smaller	smaller	decreased	all curved	curved

Gleevec	50 μM	normal	normal	normal	4/40 short	straight

AD28	50 μM	normal	normal	normal	5/28 slightly curved	straight

AD94	50 μM	Smaller	smaller swollen	Decreased	all slightly curved	curved

AD95	50 μM	smaller	normal	Decreased	all slightly curved	bent

AD115	10 μM	normal	normal	decreased around swim bladder	11/30 curved	straight

Both AD94- and AD95-treated embryos showed defective phenotypes (Fig 3). While defective phenotypes were most obvious at the later stages, they were not lethal, and embryos were alive even at day 7. For AD94, embryos showed a smaller body size, smaller puffy eyes, a minor dorsally curved tail, and less pigmentation. For AD95, all fish displayed a bent trunk (1 embryo had a severely bent trunk), slightly curved tails, smaller body size, normal eyes, craniofacial defects, and a lighter pigmentation. For AD95-treated embryos, 1 out of 9 assessed zebrafish bodies showed signs of edema. However, AD95-treated embryos showed normal V-shaped somites similar to wild-type embryos, which was in contrast to DAPT-treated embryos that completely lacked V-shaped somites (Fig. 3B).

Embryos treated with 10 μM AD115 showed a slightly abnormal phenotype and variable defects. Overall, 8 out of 30 fish had edema at 2 dpf, and one embryo displayed a bent trunk (data not shown). Among 17 embryos examined at 4 dpf and 6 dpf, 2 embryos had severe craniofacial abnormalities and did not survive beyond 6 dpf, 5 embryos showed tumors in the gut area, and 9 embryos showed craniofacial defects. Normal V-shaped somites were found among all treated embryos, and most embryos showed slightly decreased pigmentation throughout the body and especially around the swim bladder. The detailed characterization and comparison of these phenotypes are listed (Table 2) and used as reference for subsequent analysis of images automatically acquired by In Cell Analyzer.

### D. Automatic image acquisition of compound treated embryos by In Cell analyzer

**Figure 4:**
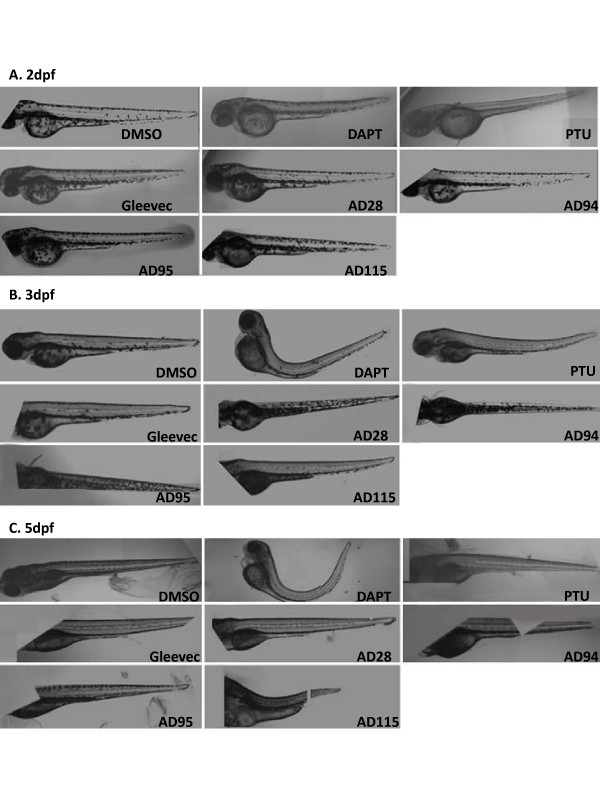
**Automatically acquired images of embryos treated with GSI or fragments of Gleevec during early development stage (6 hpf)**. Embryos were treated with GSIs or Gleevec fragments at the following concentrations at 6 hpf: 0.1%DMSO, 50 μM for all compounds, except for 10 μM AD115. Images of living zebrafish in microtiter plates were acquired by In Cell Analyzer at 2 dpf (A), 3 dpf (B) and 5 dpf (C), and several fields of images from the individual wells were automatically merged. Because the instrument acquired several fields that covered a portion of the microtiter plate well, some images show truncations of the zebrafish.

In addition to conventional microscope imaging, compound-treated embryos at 6 hpf were imaged by In Cell Analyzer (Fig. 4). This instrument is commonly used to perform high-throughput image acquisition of cultured cells living in 96- or 384-well plates. We adjusted the instrument for image acquisition of live zebrafish in microtiter plates. Upon high speed image acquisition by the instrument, several fields of images from the individual wells were automatically merged using "Merge" command from the Adobe Photoshop Elements. Most composed images contain the entire zebrafish body, but some images have truncations due to the nature of automatic image acquisition (Fig. 4). Because In Cell Analyzer did not capture the entire population of embryos in all wells, the number of analyzed zebrafish images was lower than the number of fish treated with compounds.

**Figure 5:**
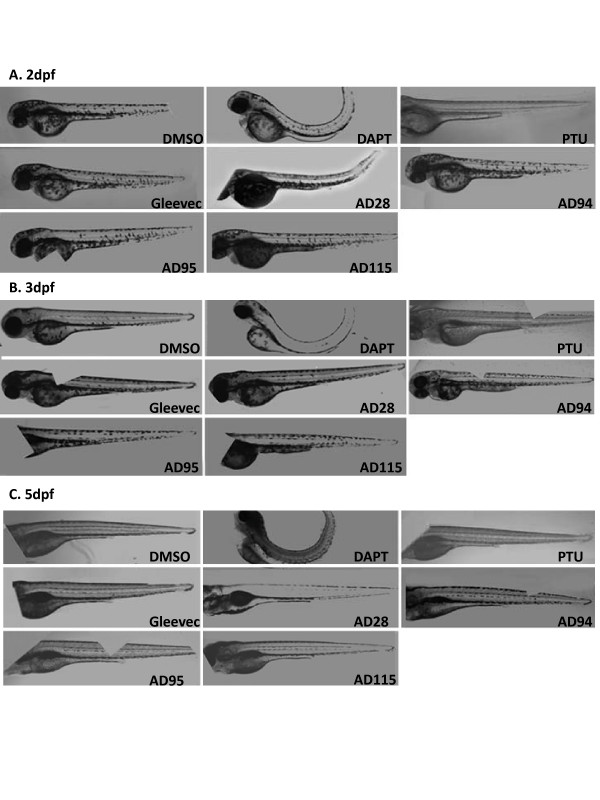
**Automatically acquired images of embryos treated with GSI or fragments of Gleevec at 24 hpf**. Embryos were treated with GSIs and fragments of Gleevec at 24 hpf, and 50 μM of all compounds, except for 10 μM of AD115, were applied to these embryos. Images of living zebrafish in microtiter plates were acquired by In Cell Analyzer at 2 dpf (A), 3 dpf (B) and 5 dpf (C), and the same procedures were followed to automatically merge several fields of images to compose the entire zebrafish.

In addition, we de-chorionated embryos at 24 hpf, transferred them to 96- or 384-well plates and treated individual embryos with different compounds. Images of the fish at 2, 3 and 5 dpf were acquired by In Cell Analyzer and the phenotypes recorded (Fig. 5). No striking difference in phenotypes between embryos treated at 6 hpf (Fig. 4, Table 3) and 24 hpf (Fig. 5, Table 4) was observed. Images of DAPT or Gleevec-treated embryos automatically acquired by In Cell Analyzer at 2 dpf and 3 dpf were similar to those recorded by conventional microscope. At 5 dpf or 6 dpf, lateral images were difficult to acquire since most fish maintained a dorsal orientation due to the development of the swim bladder.

**Table 3:** Phenotypic description of the embryos treated at 6 hpf followed by automatic image acquisition.

Cpd	Embryo Number	Conc.	Pigment	Tail	Trunk
DMSO	15	0.1%	normal	all straight	all straight

DAPT	18	50 μM	decreased	5/18 curved	10/18 curved

Gleevec	17	50 μM	normal	all straight	1/17 short

AD28	17	100 μM	normal	all straight	4/17 short

AD94	12	50 μM	decreased	all straight	all straight

AD95	10	50 μM	normal	6/10 curved	6/10 bent

AD115	15	10 μM	decreased around swim bladder	2/15 curved	all straight

PTU	16	200 μM	decreased	all straight	all straight

**Table 4:** Phenotypic description of the embryos treated at 24 hpf followed by automatic image acquisition.

Cpd	Embryo Number	Conc.	Pigment	Tail	Trunk
DMSO	16	0.1%	normal	all straight	all straight

DAPT	14	50 μM	decreased	9/14 curved	9/14 curved

Gleevec	17	50 μM	normal	all straight	all straight

AD28	11	100 μM	normal	1/11 slightly curved	1/11 short

AD94	6	50 μM	3/6 lighter	all straight	all straight

AD95	8	50 μM	normal	5/8 bent	1/8 edema

AD115	9	10 μM	decreased around swim bladder	2/9 curved	5/9 edema

PTU	11	200 μM	Decreased	all straight	all straight

The effects of compounds on pigmentation and embryo morphology, especially the tail and trunk, were readily observed. DAPT-treated embryos showed defects in tail curvature, pigmentation, eyes, and somite differentiation. Gleevec-treated embryos showed no phenotypic abnormalities. Of the Gleevec fragments, AD28-treated embryos showed no effect at 50 μM. AD94 caused a slight reduction in pigmentation, and AD115 at 10 μM decreased pigmentation around the swim bladder and caused curvature in the tails of several zebrafish. AD95 showed strong inhibition of Aβ production and caused some phenotypes in zebrafish. Phenyl thiourea (PTU) is widely used to prevent pigmentation during embryonic development for observing transparent zebrafish anatomy. As expected, all of embryos that were treated with PTU showed decreased pigmentation (Table 3 and 4).

Zebrafish treated with 50 μM of AD95 did not yield alteration in pigmentation. However, it caused curvature of tails with bent trunks. In embryos treated with 50 μM of AD95 at 24 hpf, a small number of embryos showed edema. This was not observed in embryos treated with 50 μM of DAPT. Apparently, embryos treated with AD95 and DAPT at concentrations that effectively reduced Aβ production (Fig. 1B, C) show quite different phenotypes, such as the pigmentation (Table 3 and 4) and V-shaped somites (Table 2), as described earlier.

### E. Dose-dependent phenotypic changes in zebrafish treated with increasing concentration of compounds

**Figure 6:**
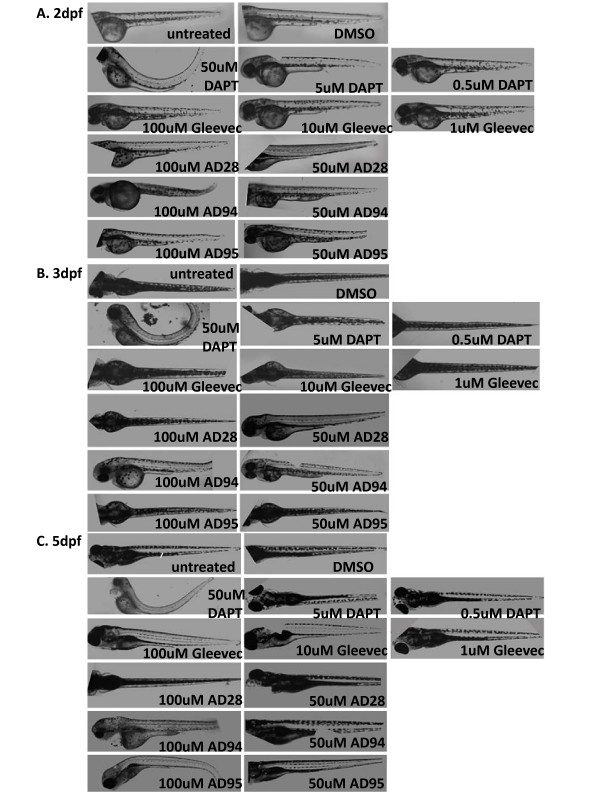
**Automatically acquired images of embryos treated with increasing concentration of GSI or fragments of Gleevec**. Images of embryos at 2 dpf (A), 3 dpf (B), and 5 dpf (C) were presented. Untreated embryos and 0.1% DMSO-treated embryos displayed similar phenotypes. Embryos treated with 50 μM DAPT showed a strong phenotype, and much less effect was observed at lower concentrations (5 μM and 0.5 μM). Gleevec was assessed at 100 μM, 10 μM, 1 μM, and embryos showed no phenotypic alteration. Embryos treated with 100 μM of AD95 showed morphologic changes. These changes were not obvious when 50 μM AD95 was used.

To search for a possible dose-dependent effect, a greater number of embryos were treated with each compound with two or three different concentrations (Table 5). Embryos at 24 hpf (Fig. 6) were treated with these compounds and In Cell Analyzer was used to capture images of embryos at 2 dpf (Fig. 6A), 3 dpf (Fig. 6B) and 5 dpf (Fig. 6C). DMSO- and untreated-embryos were used as a control. At 50 μM, DAPT-treated embryos showed tail curvature and decreased pigmentation. A minor reduction in pigmentation was also observed in embryos treated with 5 μM DAPT, but no defects in tail curvature or trunk formation were detected at the lower concentration. The dose-response phenotypes acquired by In Cell Analyzer were similar to those collected by conventional microscope [40]. The majority of Gleevec-treated embryos showed no defects in morphology, even at the highest concentration of 100 μM. At this concentration, a decrease of pigmentation was observed in embryos treated at 24 hpf (Table 5). AD28 and AD94 did not cause much difference in phenotypes when two different concentrations of compounds were used. AD115 was not tested since it was lethal at doses higher than 10 μM.

**Table 5:** Dose-dependent phenotypic alteration in the embryos treated at 24 hpf.

Cpd	Embryo Number	Conc.	Pigment	Tail	Trunk
DMSO	11	0.1%	normal	all straight	all straight

DAPT	11	50 μM	decreased	11/11 curved	11/11 curved

DAPT	9	5 μM	decreased slightly	all straight	1/9 deformed

DAPT	4	0.5 μM	normal	all straight	all straight

Gleevec	9	100 μM	decreased	all straight	all straight

Gleevec	7	10 μM	normal	1/7 curved	1/7 short

Gleevec	7	1 μM	normal	all straight	all straight

AD28	14	100 μM	decreased slightly	all straight	all straight

AD28	4	50 μM	normal	all straight	all straight

AD94	8	100 μM	lighter	all edema	all edema

AD94	4	50 μM	lighter	all straight	all straight

AD95	7	100 μM	lighter	all curved	all bent, 5/7 edema

AD95	4	50 μM	normal	all bent	all bent, 3/4 edema

Untreated	3		normal	all straight	all straight

While no effect on pigmentation of AD95 at 50 μM was observed, a minor reduction in pigmentation was detected when 100 μM of AD95 was used to treat embryos at 24 hpf (Table 5). The alterations in tails and trunks were more severe at the high dose of AD95. Generally, most phenotypes observed in these embryos were specific and correlated well with the dose of DAPT, Gleevec and its fragments.

To investigate the mechanistic side effects associated with GSIs and Gleevec fragments and to explore the Notch signaling that may contribute to these phenotypes, the expression of the Notch downstream target gene *her6 *was examined by *in situ *hybridization and the intensity of *her6 *expression to the effect of inhibitors, i.e., a loss of *her6 *staining that corresponds to an inhibition of γ-secretase-mediated Notch signaling, were compared. This approach has been used successfully by us and others to measure γ-secretase cleavage of Notch in zebrafish [5,41].

**Figure 7:**
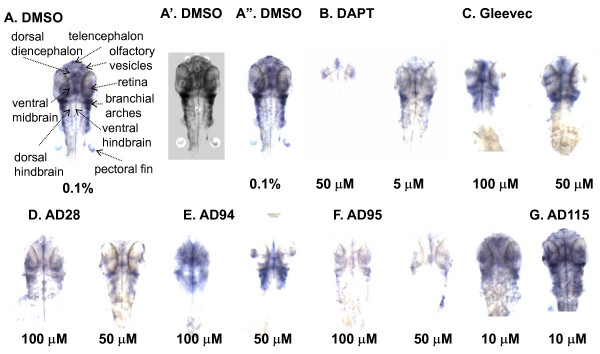
**Effect of GSI and fragments of Gleevec on Notch target gene *her6 *expression**. Embryos were treated with the compounds at 24 hpf and fixed at 48 hpf in 4% formaldehyde. *In situ *hybridization was performed to stain *her6 *staining. In control embryos, *her6 *expression was abundant in the dorsal diencephalon, retinas, ventral midbrain and ventral hindbrain, and it was lower in telencephalon, olfactory vesicles, branchial arches, ventral hindbrain and pectoral fins A, these regions are indicated by arrows; A', the *her6*-positive brain regions from the dorsal view images were manually segmented; A", the same embryo was illustrated for the comparison of staining intensity. B-G, *her6 *expression in embryos treated with different concentrations of DAPT, Gleevec, or fragments of Gleevec.

In DMSO-treated control embryos, *her6 *expression was abundant in the dorsal diencephalon, retinas, ventral midbrain and ventral hindbrain (Fig. 7A). The expression of *her6 *was also observed in telencephalon, olfactory vesicles, branchial arches, ventral hindbrain and pectoral fins (Fig. 7A). The pattern of *her6 *staining is consistent with previous reports [5]. In the presence of 50 μM DAPT, the *her6 *expression was lost in most areas, demonstrating a complete inhibition of γ-secretase activity in the animal (Fig. 7B). The residual γ-secretase activity might remain in olfactory vesicles and telencephanlon where weak staining of *her6 *was detected (Fig. 7B). When embryos were treated with a much lower concentration of DAPT at 10 μM, weaker staining of *her6 *was observed in most areas found in DMSO-treated embryos, with almost identical staining of *her6 *in ventral midbrain (Fig. 7B). Treatment of embryos with Gleevec (Fig. 7C) and AD28 (Fig. 7D) caused a slight reduction of *her6 *staining at 100 μM, and the effect was much weaker when 50 μM of compounds were used. The staining of *her6 *in embryos treated with 50 and 100 μM AD94 (Fig. 7E) was similar to those treated with Gleevec and AD28. AD115 did not have any effect on *her6 *expression and showed identical pattern to control embryos (Fig. 7G).

AD95 treated embryos showed a regionally specific, dose-dependent inhibition of *her6 *expression (Fig. 7F). At 10 μM, AD95 did not reduce much of *her6 *staining, compared to DMSO-treated embryos. When embryos were treated with 50 μM or 100 μM of AD95, *her6 *staining was no longer detected in most areas, except for four regions. No difference in *her6 *staining in retina and branchial arches was observed between embryos treated with 50 μM and 100 μM AD95, but the dorsal diencephalon and ventral midbrain in embryos treated with 100 μM had weaker staining than those treated with 50 μM AD95. The intensity of staining in these four areas from AD95-treated embryos was reduced compared to DMSO-treated embryos.

**Figure 8:**
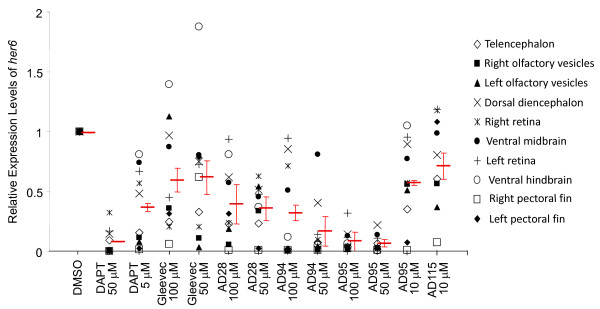
**Quantification of Notch target gene *her6 *expression in embryos treated with GSI or fragments of Gleevec**. Based on the segmented region of interest as highlighted in Fig. 7 A', the intensity of each ROI was quantified. The levels of *her6 *expression in DMSO treated embryos were normalized to 1, and relative levels of other embryos were calculated. Different regions of embryos were quantified, and the average of staining intensity of embryos treated with an individual compound was presented by a horizontal line in red, and the bar represents the standard error of means.

To quantify the intensity of her6 staining, we highlighted the regions of interest (Fig 7A') that corresponded to unique brain structures (Fig. 7A). We calculated the intensity of all regions of interest and obtained the average levels of her6 expression in these embryos (Fig. 8). Overall, there was a reduction of her6 staining in all drug-treated embryos, with DAPT and AD95 showed most effects. To determine whether there was direct effect of these compounds on γ-secretase complex, we used in vitro γ-secretase activity assay to examine the Notch cleavage. Using N100 as substrate, we treated partially solubilized γ-secretase with different compounds at 100 μM. While we found most compounds did not have any effect on Notch cleavage in vitro, AD 28. AD115 and DAPT showed inhibition of Notch cleavage (Table 6).

**Table 6:** In vitro inhibition of Notch cleavage by AD28 and AD115.

Cpd	Conc.	In vitro Inhibition of Notch Cleavage
DMSO	0.10%	-

DAPT	100 μM	+

Gleevec	100 μM	-

AD28	100 μM	+

AD94	100 μM	-

AD95	100 μM	-

AD115	100 μM	+

## Discussion

The strength of image acquisition of living zebrafish in 96-and 384-well plates is fast data collection since no manual intervention was necessary for image recording. More importantly, the images obtained were a high quality that is comparable to those images captured by a conventional microscope. Instead of using transgenic zebrafish that express green fluorescent protein, wild type zebrafish embryos were routinely treated with different drugs and bright field images were acquired. For the first time, images of living zebrafish arrayed in a microtiter plate were acquired uniformly. The use of microtiter plate is an industrial standard for in vitro assays, such as enzymatic assays or cell-based metabolic assays.

Matlab and Adobe Photoshop Elements software were used for the composition and processing of automatically acquired images with minimum manual intervention. Due to the size of zebrafish embryos and the resolution that is required for phenotype analysis, the difficulties in capturing the entire embryo in 96- or 384-well plates reflect a primary weakness of this approach. In this regard, housing zebrafish embryos in 384-well plates is better because less blank space is left in the images. Furthermore, compared to images of zebrafish housed in 96-well plates, a higher number of images contained a portion of zebrafish embryos living in 384-well plates, which allows easier image processing by algorithms. It was noticed that parts of the zebrafish images were still missing after automatic composition as shown in several figures. This problem was approached by using more embryos and extracting essential biological information from these images that were composed and processed by Matlab and Adobe Photoshop Elements. Further development should be pursued to make this widely useful for drug screening in zebrafish.

Zebrafish is an ideal vertebrate for primary toxicity studies in whole animals because of their cost-effectiveness, the ease of drug delivery, and their high sensitivity to toxins. Among all compounds at the tested concentrations, apoptosis was observed within a few cells. However, the effect was not massive and was comparable to the DMSO-treated embryos. In two independent experiments, AD94 showed a slightly higher apoptotic phenotype in the tail region than AD95 and DMSO-treated embryos. However, DAPT- and AD95-treated embryos showed defects in the tail and trunk development, but had fewer apoptotic cells than AD94. The occurrence of apoptosis in the tail region may not be the cause for morphological changes in the trunk since this effect was observed with all compounds tested. Therefore, perhaps compound-produced defects are more mechanistic toxicities induced by the blockage of specific pathways, e.g., an inhibition of Notch signaling rather apoptosis.

The simplification of high throughput drug screening in whole animals was illustrated in this test of zebrafish that were treated with different concentrations of various compounds. Although we had only used a few concentrations in this study, this limitation could be resolved in the future when more concentrations are tested for potential lead compounds. The number of images varied among compounds, depending on fish survival, positioning, and timing of photo acquisition. Images taken at earlier stages, such as 2 or 3 dpf, were usually more informative since embryos were immobile and both dorsal and lateral views could be obtained. At the later stages, sometimes only dorsal view can be imaged, as zebrafish mainly maintain a dorsal position. Nevertheless, we have successfully obtained high-quality images of zebrafish growing in 384 well plates, and the advance we have achieved is not only the quality of current zebrafish images but also the quantity of potential images we can acquire in a short period. The scope of developing a complete set of techniques to automatically acquire living zebrafish in a 384 well plates evolves with the advance of optical instruments and computing power. The current set of techniques allow a bench scientist to perform drug screening in a living vertebrate, and further automation could be developed, e.g., applying robotics for liquid handling. Exploration of this technique should be pursued to make it widely useful for drug screening in vertebrate animals.

In this study, phenotypes of zebrafish treated with known γ-secretase inhibitors, DAPT, Gleevec, and its fragments were examined. DAPT-treated embryos showed most defects in tail curvature, pigmentation and craniofacial structures as previously reported [42]. Over 90% of the Gleevec-treated embryos displayed a wild-type phenotype, and a few embryos showed a short, but not curved tail. One of the smaller fragments of Gleevec, AD28, has the least effect in all of the embryonic studies performed. This may be due to the simplicity of its structure such that AD28 may not have key moieties or interactions necessary for subsequent findings observed with Gleevec and AD95.

Fragment AD115 lacked Aβ inhibition and caused mortality of all fish at 50 μM. Lowering the concentration of AD115 to 10 μM also produced embryonic defects, such as pigmentation defects around the swim bladder and defects in craniofacial development. Also, even at this lower concentration, bumps around the gastrointestinal tract were observed. These results suggest that AD115 has significance differences in its structure from both Gleevec and AD95 such that treatment of zebrafish embryos with AD115 yield undesirable effects versus the overall results observed with both Gleevec and AD95. These differences in findings indicate that there are compounds within a chemical series or analogs thereof that could set the stage for identifying improved candidate compounds as potential therapeutic agents.

The results indicate that AD95 clearly reduces Aβ production but also affects the morphology of zebrafish embryos as does AD94 and AD115. If compounds such as AD95 were to be pursued as potential GSIs as a possibly therapy for the treatment of AD, Notch processing would have to be examined in much details, and AD95 and analogs need to be further explored for selectively inhibiting APP processing. Since APP and Notch are competitively processed by γ-secretase [43], it is essential to selectively inhibit the cleavage of APP substrate and not Notch. It is interesting that AD95-treated embryos showed normal V-shaped somites unlike DAPT-treated embryos. Perhaps DAPT and AD95 block γ-secretase differently and perturb unrelated downstream pathways which are partly illustrated by somitogenesis, another interesting facet of this research to further explore. While Gleevec, AD28 and AD94 showed slight reduction of *her6 *staining, their phenotypes were not completely identical. An animal treated with compounds usually show many phenotypes, and some of them are related to Notch signaling. We do not expect that all phenomenon found in these fish are caused by altered Notch signaling, as other metabolic pathways might be changed in the presence of these compounds. Similarly, several compounds showed different potencies in vitro and in vivo, strongly suggesting that additional components are involved in mediating the effect of GSI and fragments of Gleevec.

## Conclusion

Two methods were used to acquire images of zebrafish embryos that were treated with DAPT, Gleevec, and fragments of Gleevec, the conventional microscope imaging and a new semi-automated image acquisition method. Easy compound delivery and good resolution of images result in the detailed examination of the effects of potential drugs on the phenotype of zebrafish. Hence, this approach provides a platform for a high-throughput screen of a library of compounds in a whole animal and establishes the zebrafish as a model vertebrate for screening therapeutic agents for AD and other diseases.

## Competing interests

The authors declare that they have no competing interests.

## Authors' contributions

DA and TY carried out biochemical and zebrafish experiments, XX designed algorithms for image processing, STW participated in the study design, CEA provided compounds for zebrafish treatment, WX designed the study, DA and WX wrote the manuscript. All authors read and approved the manuscript.

## Abbreviations

AD: Alzheimer's disease; Aβ: amyloid β protein; Abl: Abelson leukemia; dpf: days post fertilization; GSI: γ-secretase inhibitor; hpf: hours post fertilization; NICD: Notch intracellular domain.
